# Walnut supplementation after fructose-rich diet is associated with a beneficial fatty acid ratio and increased ACE2 expression in the rat heart

**DOI:** 10.3389/fphys.2022.942459

**Published:** 2022-09-21

**Authors:** Maja Bošković, Maja Živković, Goran Koricanac, Snezana Tepavcevic, Manja Zec, Jasmina Debeljak-Martacic, Aleksandra Stanković

**Affiliations:** ^1^ Laboratory for Radiobiology and Molecular Genetics, “VINČA” Institute of Nuclear Sciences—National Institute of the Republic of Serbia, University of Belgrade, Belgrade, Serbia; ^2^ Laboratory for Molecular Biology and Endocrinology, “VINČA” Institute of Nuclear Sciences—National Institute of the Republic of Serbia, University of Belgrade, Belgrade, Serbia; ^3^ School of Nutritional Sciences and Wellness, University of Arizona, Tucson, AZ, United States; ^4^ Centre of Excellence in Nutrition and Metabolism Research, Institute for Medical Research, National Institute of Republic of Serbia, University of Belgrade, Belgrade, Serbia

**Keywords:** walnuts, fructose-rich diet, omega-3 PUFA, heart, renin-angiotensin system, NF-κB

## Abstract

Increased fructose consumption has been linked with chronic inflammation and metabolic syndrome (MetS). Activation of the renin-angiotensin system (RAS) and NF-κB have been detected in MetS. Walnuts are a rich source of polyunsaturated omega-3 fatty acids (n-3 PUFA) that were suggested to exert anti-inflammatory effects related to cardio-metabolic health. We hypothesized that walnut supplementation has the capacity to revert unfavorable fructose-rich diet (FRD)-induced activation of cardiac RAS and NF-κB in male rats. Due to the lack of similar studies, we investigated the effects of walnut supplementation (6 weeks) on the expression of four RAS molecules (ACE, ACE2, AT1R, and AT2R) and NF-κB in rat heart after FRD (10% w/v, 9 weeks). In addition, we followed the changes in the n-6/n-3 PUFA ratio in the total pool of heart lipids after both treatments to elucidate the walnut effects on fatty acids in the heart. 36 animals (9 per group) participated in the experiment. FRD significantly increased the ACE protein level in the heart (*p* < 0.001). Walnut supplementation significantly increased the ACE2 protein level in the heart of FRD (*p* < 0.001). In addition, walnut supplementation showed a significant main effect on the arachidonic acid/eicosapentaenoic acid ratio (*p* = 0.004). Walnut supplementation significantly reduced this ratio, in comparison with both, the control group (C vs. FW, *p* < 0.05) and the FRD group (F vs. FW, *p* < 0.05). However, walnut treatment failed to revert the significant effect of fructose (*p* < 0.001) on the elevation of NF-κB protein level. Our results suggest a beneficial effect of walnut supplementation on ACE2 protein level and n-6/n-3 PUFA level in the heart of the animal model of MetS. Such results highlight the approach of omega-3-rich walnut supplementation in the stimulation of endogenous production of favorable molecules in the heart which could be an affordable nutritional treatment formaintenance of cardio-metabolic health.

## Introduction

Metabolic syndrome (MetS) has been correlated with increased fructose consumption over the past several decades (Taskinen et al., 2019). Previously, activation of the renin-angiotensin system (RAS) has been highlighted to be important in the development of MetS (Sowers, 2004; Bundalo et al., 2016). Cardiac RAS, which can be activated independently of systemic RAS, has a role in the regulation/modulation of cardiac and coronary function, apoptosis, inflammation, metabolism, growth, and heart remodeling.

Angiotensin-converting enzyme (ACE) and ACE homologue (ACE2) are the main enzymes of RAS. ACE2 is a negative regulator of RAS, counterregulating ACE production of angiotensin II (Ang II) in target tissues.

The effects of Ang II are primarily mediated by two G-protein coupled receptors, angiotensin II type 1 receptor (AT1R) and angiotensin II type 2 receptor (AT2R), which achieve different physiological effects by activating different signaling pathways (Levy, 2004). AT1R activates pathways related to cell growth and proliferation, inducing harmful effects of Ang II, such as oxidative stress, endothelial dysfunction, and inflammation (Mehta and Griendling, 2007). Activation of AT2R causes opposite effects, such as vasodilation and hypotension, inhibition of cell growth and proliferation by promotion of apoptosis, and possible inhibition of AT1R, acting as an AT1R antagonist (Levy, 2004; Savoia et al., 2011; Wang et al., 2012). AT2R gene expression can be stimulated in pathological conditions, i.e. after tissue damage (Steckelings et al., 2010). AT2R stimulation counteracts many of the AT1R-mediated actions in the presence of a selective AT1R antagonist and may potentiate the effects of angiotensin receptor blockers (Savoia et al., 2011). The study of Savoia et al. demonstrated the functional contribution of AT2R-mediated vasodilation to the antihypertensive effects of selective AT1R blockade in high cardiovascular risk patients (Savoia et al., 2007).

AT1R-mediated activation of nicotinamide adenine dinucleotide phosphate (NADPH) oxidase results in the production of reactive oxygen species (ROS) causing oxidative stress which promotes inflammation, by activation of nuclear factor kappa B (NF-κB) and transcription of proinflammatory cytokine genes (Wang et al., 2012; Nowotny et al., 2015). It affects the insulin signaling pathway leading to insulin resistance (Zhang et al., 2008). These data link RAS, oxidative stress, and inflammation as central players in the insulin signaling pathway underlying metabolic disturbances in MetS (Nelson and Bremer, 2010).

After the characterization of ACE2, its functional role in the heart was somewhat controversial. However, the findings reported by Mercure et al. (2008) shed new light on the beneficial actions of the ACE2/Ang (1-7) axis within the heart, noting that the cardioprotective effects of ACE2 result from a direct degradation of Ang II and inhibition of AT1R-mediated signaling. Recent studies underlie the anti-inflammatory and anti-fibrotic roles of ACE2 in the kidney and heart, as potential therapeutic targets to complement known effects of ACE inhibitors and AT1R blockers (reviewed in Simões E Silva and Teixeira, 2016). After myocardial infarction, ACE2 deficient hearts increased the production of ROS and activated inflammatory cascade (Kassiri et al., 2009). Many studies have reported that ACE2/Ang (1-7) axis attenuates the manifestations of MetS by reducing the amount of fat tissue and plasma lipid levels and enhancing glucose tolerance and insulin sensitivity (Santos et al., 2010; Liu et al., 2012).

In conditions of cardiac RAS activation, such as MetS, the role and augmentation of ACE2 have been stressed out. The approach of increasing endogenous ACE2, particularly using natural compounds, is morefavourable than providing an exogenous one using human recombinant or adenoviral ACE2 in animal disease models.

Recently, the protective effects of walnut (Juglans regia L.) supplementation were detected in male rats following the FRD-induced decrease in antioxidative capacity of cardiac tissue and increase in plasma predictors of low-grade inflammation (Bošković et al., 2021). Walnuts are a rich source of polyunsaturated omega-3 fatty acids (n-3 PUFA) that are known to have beneficial effects in the prevention of cardiovascular and metabolic diseases (Scott et al., 2017; Oppedisano et al., 2020). The anti-inflammatory effect of walnut fatty acids, expressed in the form of a decreased n-6/n-3 ratio (Zec et al., 2020; Bošković et al., 2021), is likely mediated through the negative regulation of NF-κB signaling. Indeed, it has been shown that increased arachidonic acid (AA, 20: 4n-6)/eicosapentaenoic acid (EPA, 20: 5n-3) ratio is directly associated with an increased risk of cardiovascular (CVD) and MetS-related diseases (Ninomiya et al., 2013; Takahashi M et al., 2017) and represents an inflammatory biomarker in these disorders (Tutino et al., 2019).

Therefore, we hypothesized that walnut supplementation had the capacity to revert unfavorable FRD-induced activation of cardiac RAS and NF-κB in male rats. Totest our hypothesis, we investigated the RAS-mediated inflammatory effects of FRD (10% w/v, 9 weeks) and anti-inflammatory effects of walnut supplementation (6 weeks) based on the expression of four RAS molecules (ACE, ACE2, AT1R, and AT2R) and NF-κB in rat heart. Based on the relationship between the n-6/n-3 ratio and inflammation supported by our previous findings in plasma (Bošković et al., 2021) regarding fructose and walnut supplementation effects on AA/EPA and AA/docosahexaenoic acid (DHA, 22: 6n-3), we hypothesized that walnut-enriched diet should also have the capacity to restore beneficial ratios of these PUFAs in the heart.

## Materials and methods

### Animal model and treatment

The animal protocols were approved by the Ethical Committee of the “Vinča” Institute of Nuclear Sciences for the Use of Laboratory Animals and performed in accordance with the guidelines of Directive 2010/63/EU of the European Parliament. Male Wistar rats, twenty-one-day-old, were divided into two groups. The control (C, *n* = 18) and fructose-fed (F, *n* = 18) groups were fed normal rat chow and drank water and 10% (w/v) fructose solution, respectively. Three rats per cage were housed under standard temperature (22°C) and 12 h light/dark cycles. After 9 weeks of this diet regime, half the number of animals from each group received daily 2.4 g of dietary walnuts in form of whole kernels (CW, *n* = 9, FW, *n* = 9) for 6 weeks. The complete fatty acid, macronutrient, and mineral composition of the walnuts (Juglans regia L.) have been described by arecent study (Petrović-Oggiano et al., 2020). The animals were euthanized, hearts were removed and stored at −70°C until Western blot, quantitative reverse transcription–polymerase chain reaction (qRT-PCR), and fatty acid analysis in the total pool of heart lipids. 9 animals per group (a total of 36 animals) pooled in 3 samples (each consisting of 3 animals of the same group) participated in the experiment, corresponding to the standards of statistics and ethics.

### Fatty acid analysis in the total pool of heart lipids

Total lipid content in the heart was isolated by a modified method of Harth and collaborators (Harth et al., 1978). The hearts were crushed with a pestle in three types of solvents −5 ml chloroform (chl): methanol (met) with 10 mg % BHT (2:1, v/v), then 5 ml chl:met:5% water (1:1, v/v) and finally 5 ml chl:met (1:1, v/v). The crushed hearts were left in the freezer for 24 h. The extracts were filtered and washed out with 3 ml of each type of solvent system, then evaporated using a vacuum evaporator and resuspended in 300 µl chl:met (2:1, v/v).

Fatty acids were esterified into methyl esters prepared by transmethylation with 3 N HCl in methanol at 85°C for 1 h, then reconstituted in hexane and separated by gas chromatography using a Shimadzu chromatograph (GC-2014, Kyoto, Japan) equipped with a flame-ionization detector and a fused silica gel capillary column, RTX 2330 (60 m × 0.25 mm and × 0.2 μm film thickness) (Restek Co., Bellefonte, PA, United States). Methyl esters of individual fatty acids were identified by comparing the peak retention times of samples with a PUFA-2 standard mixture and the “Supelco 37 component fatty acid methyl ester mix” (Supelco, Inc., Bellefonte, PA, United States). For the purposes of this study, AA, EPA, and DHA were identified in the total pool of heart lipids. The amount of these PUFAs in the heart was expressed as a percentage of all identified fatty acids in the total pool of heart lipids.

### Cardiac lysate preparation

Lysate fraction was prepared from pooled cardiac tissue of three animals of the same group. Hearts were homogenized on ice with an Ultra-Turrax Homogenizer in modified RIPA buffer (pH 7.4) containing 50 mM Tris–HCl, pH7.4, 150 mM NaCl, 1% Triton X-100, 0.2% Na-deoxycholate, 0.2% SDS, 1 mM EDTA, pH7.4, protease inhibitors (1mM PMSF, 10 μg/ml leupeptin, and 10 μg/ml aprotinin), and phosphatase inhibitors (1mMactivated sodium orthovanadate and 10 mM sodium fluoride). The homogenates were centrifuged at 15,000 × g for 30 min at 4°C and obtained supernatants were boiled in Laemmli sample buffer. They were used as a cardiac cell lysate for Western blot analysis.

### Western blot analysis

After the preparation of cardiac lysates, the proteins (30 μg/lane) were separated by size on 10% sodium dodecyl sulphate (SDS) polyacrylamide gel by electrophoresis. The separated proteins were then transferred to polyvinylidene fluoride (PVDF) membranes. Total protein staining of the membranes with Ponceau S (Sigma-Aldrich, P3504) was used to assess the uniformity of protein loading in each lane and served as a loading control (Romero-Calvo et al., 2010). After destaining and blocking with 5% (w/v) milk in TBST, the membranes were incubated with primary antibody for ACE (sc-20791), ACE2 (sc-20998), AT1R (ab124505), AT2R (ab254561) or NF-κB [sc-372(C-20)] overnight. After washing with TBST, the membranes were incubated with a secondary anti-rabbit antibody conjugated with horseradish peroxidase (HRP) (Santa Cruz Biotechnology) for 1.5 h at room temperature. After the final washing step, the signal that reflects antigen-antibody binding was detected by the enhanced chemiluminescence (ECL) method. We run three, and two technical replicates for ACE/ACE2 and NF- κB, respectively in comparison to two blots for AT1R/AT2R. Technical replicates were run to overcome intra- and inter-blot variability or strong background. The films were scanned and densitometry analysis of protein bands of interest was performed using ImageJ software (NIH, United States). To obtain the most reliable results, and to prevent bias in selecting particular samples, we included values from all the gels in the quantification analysis. The results were expressed as the protein/total protein staining ratio.

### RNA isolation and quantitative real-time reverse transcriptase-PCR

Total RNA was extracted from the frozen (−70°C) hearts using TRIzol Reagent (Ambion, Inc.) according to the manufacturer’s instructions. The RNA was quantified spectrophotometrically (NanoDrop^®^ ND-1000 spectrophotometer, Thermo Scientific) by reading the optical density (OD) at 260 and 280 nm. Total RNA, treated with DNase I (1 U/2 μg RNA) for 1 h at 37°C, was converted to complementary DNA (cDNA) by reverse transcription. Reverse transcription was performed using 2 μg of total pure RNA and First Strand cDNA Synthesis kit, with oligo (dT) 18 primers, according to the manufacturer’s instructions (Fermentas, Lithuania).

Detection of AT1R and AT2R expression levels was done by amplification of cDNA by real-time PCR using an ABI Real-time 7500 system (ABI, Foster City, CA). All reactions were performed in duplicates, in a total volume of 25 μl using the pre-developed TaqMan^®^ Gene Expression Assays for AT1R (Rn02758772_s1, Thermo Fisher Scientific, Waltham, MA United States) and AT2R (Rn00560677_s1, Thermo Fisher Scientific, Waltham, MA United States) which included specific RT Primers and TaqMan^®^ Probes. The 18 s rRNA served as an internal reference for AT1R and AT2R gene expression, and it was detected by TaqMan^®^ Gene Expression Assay ID Hs99999901_s1 (Thermo Fisher Scientific, Waltham, MA United States). The amplification conditions included initial denaturation at 95°C for 10 min, followed by 40 cycles of denaturation at 95°C for 15 s and annealing at 60°C for 60 s. Differences in mRNA expression between groups were analyzed using the comparative 2−ΔCt method (Livak and Schmittgen, 2001) and data are expressed as relative gene expression.

### Statistical analysis

Statistica software package was used for statistical analysis. Values were expressed as mean ± standard deviation (SD) for 3 samples, each consisting of 3 pooled hearts of the same group (9 animals per experimental group, a total of 36 animals). All the values from the gels along with removing outliers were included in the quantification analysis. The majority of outliers have been removed in the analysis of proteins that required technical replicates, if the band was not clear on the blot or if the data points were varying twenty-five percent or more from the majority of data in a particular experimental group. Two-way analysis of variance (ANOVA) was used to determine the significance of fructose and walnut treatment as independent factors, as well as their mutual interactions. Tukey’s post hoc test was used to determine the significance of differences between groups. *p* < 0.05 indicated a significant difference. If the technical replicates were run the Bonferroni correction for multiple testing was applied and consequently, in those analyses, the minimal cut of value for *p* was 0.016.

## Results

### Effects of fructose-rich diet and walnut supplementation on AA/EPA and AA/DHA ratio in the total pool of heart lipids

**FIGURE 1:**
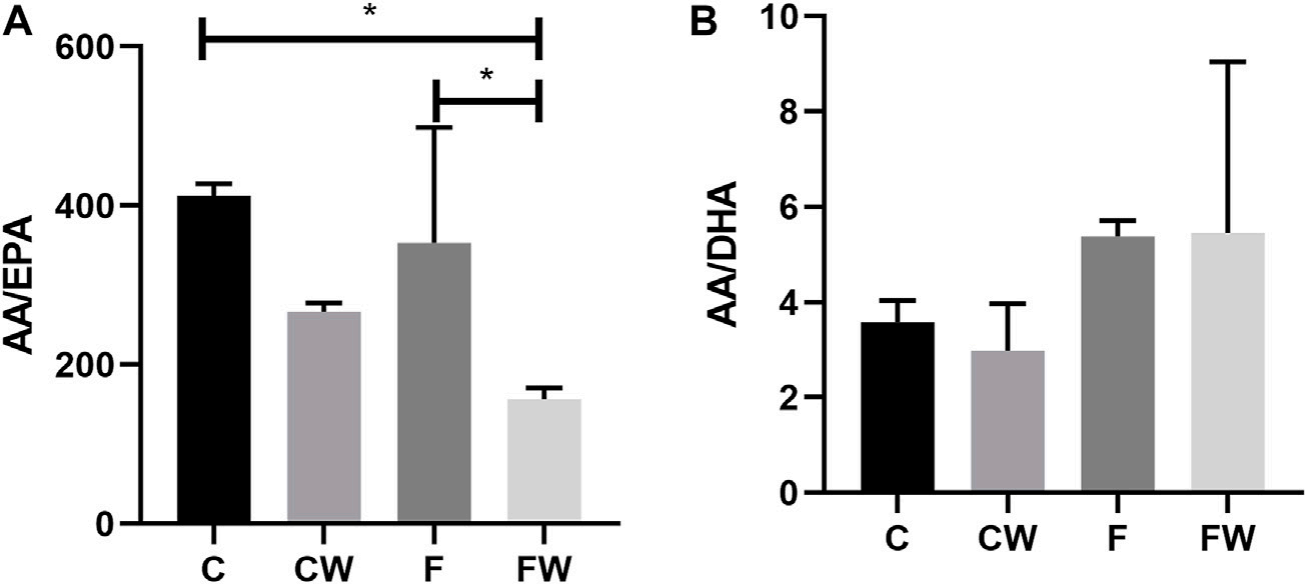
Effects of fructose-rich diet and walnut supplementation on the **(A)** AA/EPA and **(B)** AA/DHA ratio in total heart lipid pool of experimental rats. Values are means with standard deviations represented by vertical bars for 3 animals per group. C—animals on standard laboratory chow, F—animals fed a fructose-rich diet, CW—animals on standard laboratory chow and walnut supplementation, FW—fructose-fed animals on walnut supplementation, *p < 0.05.

Walnut supplementation showed a significant main effect on the AA/EPA ratio (*p* = 0.004). Walnut supplementation significantly reduced this ratio, in comparison with both, the control group (C vs. FW, *p* < 0.05) and the FRD group (F vs. FW, *p* < 0.05) (Figure 1A). Neither FRD nor walnut supplementation showed a significant main effect on the AA/DHA ratio (Figure 1B).

### Effects of the fructose-rich diet and walnut supplementation on the expression of ACE and ACE2 in the heart of male rats

**FIGURE 2:**
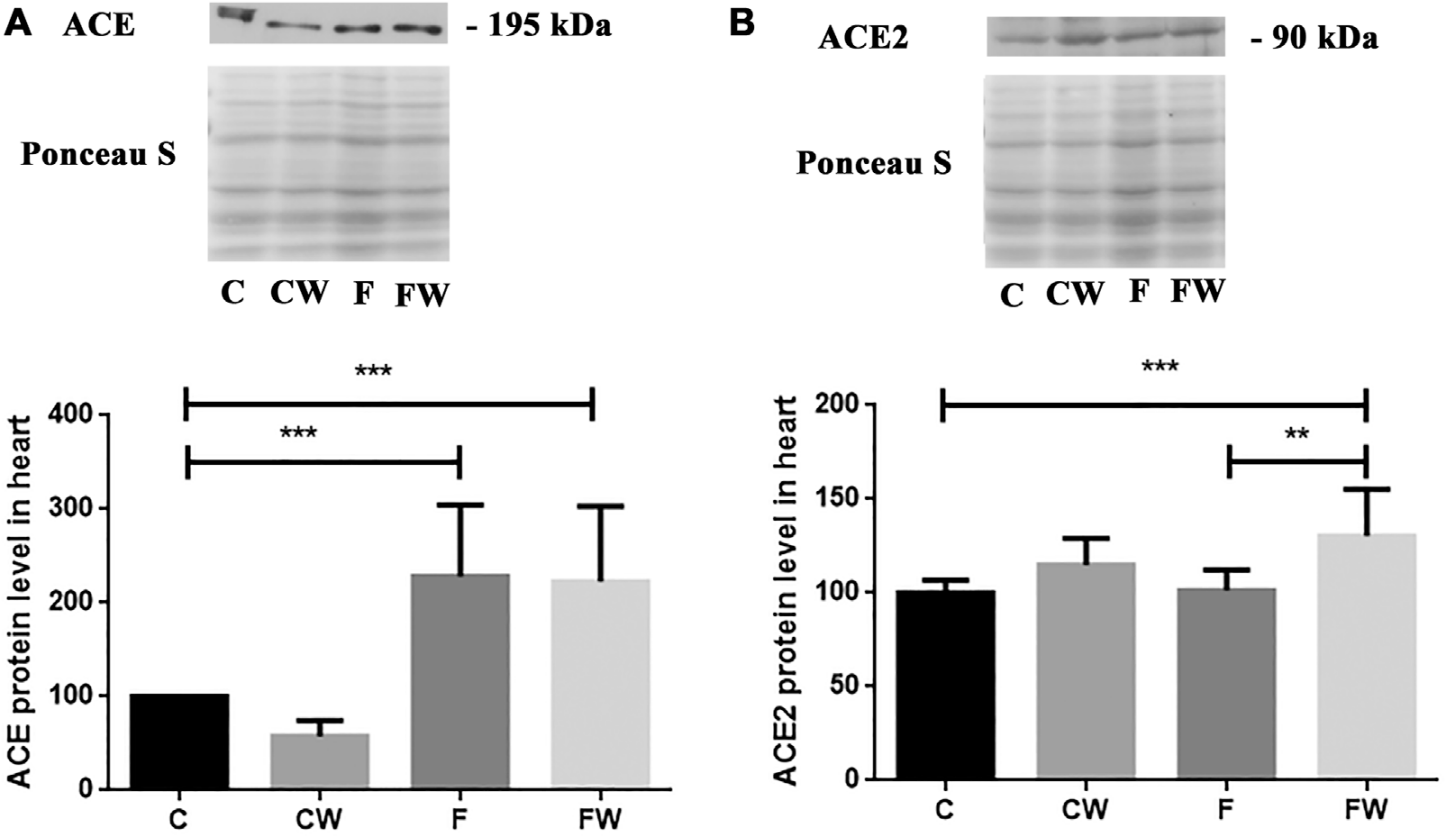
Effects of fructose-rich diet and walnut supplementation on **(A)** ACE and **(B)** ACE2 protein level in heart of experimental rats. Values are means with standard deviations represented by vertical bars for 3 samples, each consisting of 3 pooled hearts of the same group. C—animals on standard laboratory chow, F—animals fed a fructose-rich diet, CW—animals on standard laboratory chow and walnut supplementation, FW—fructose-fed animals on walnut supplementation, **p < 0.01, ***p < 0.001.

The present study showed the significant main effect of fructose (*p* < 0.001) on the ACE protein level and the significant main effect of walnut supplementation (*p* < 0.001) on the ACE2 protein level in the heart. FRD significantly increased the ACE protein level in rat hearts compared to control animals (C vs. F, *p* < 0.001) (Figure 2A), while cardiac ACE2 expression remained unchanged compared to controls (Figure 2B). Combined fructose and walnut treatment elevated both, ACE and ACE2 protein level compared to control rats (C vs. FW, *p* < 0.001, for both) and increased the ACE2 protein level compared to fructose-fed animals (F vs. FW, *p* < 0.01).

### Effects of the fructose-rich diet and walnut supplementation on the expression of AT1R and AT2R in the heart of male rats

**FIGURE 3:**
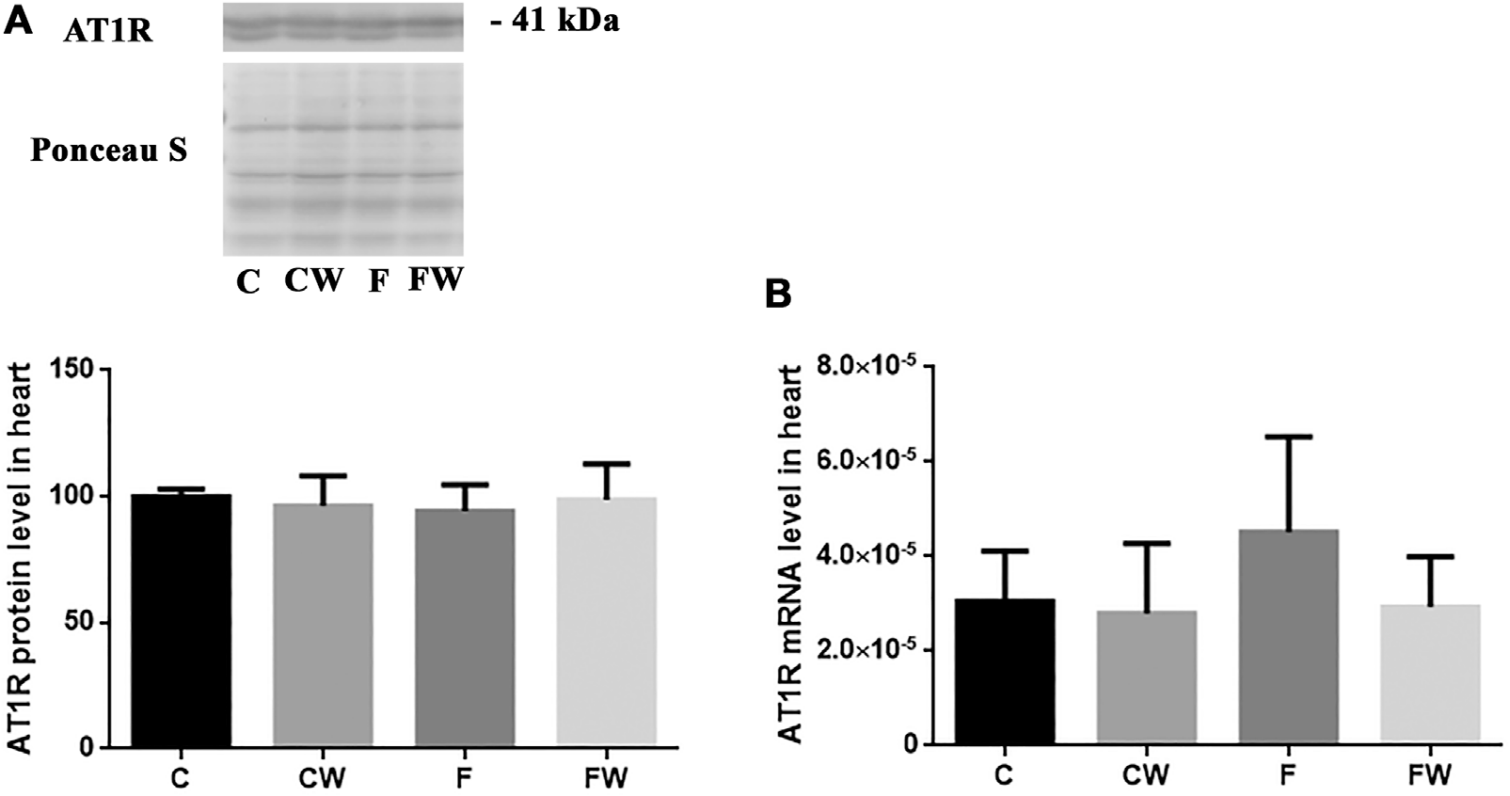
Effects of fructose-rich diet and walnut supplementation on **(A)** protein and **(B)** mRNA level of AT1R in heart of experimental rats. Values are means with standard deviations represented by vertical bars for 3 samples, each consisting of 3 pooled hearts of the same group. C—animals on standard laboratory chow, F— animals fed a fructose-rich diet, CW—animals on standard laboratory chow and walnut supplementation, FW—fructose-fed animals on walnut supplementation.

**FIGURE 4:**
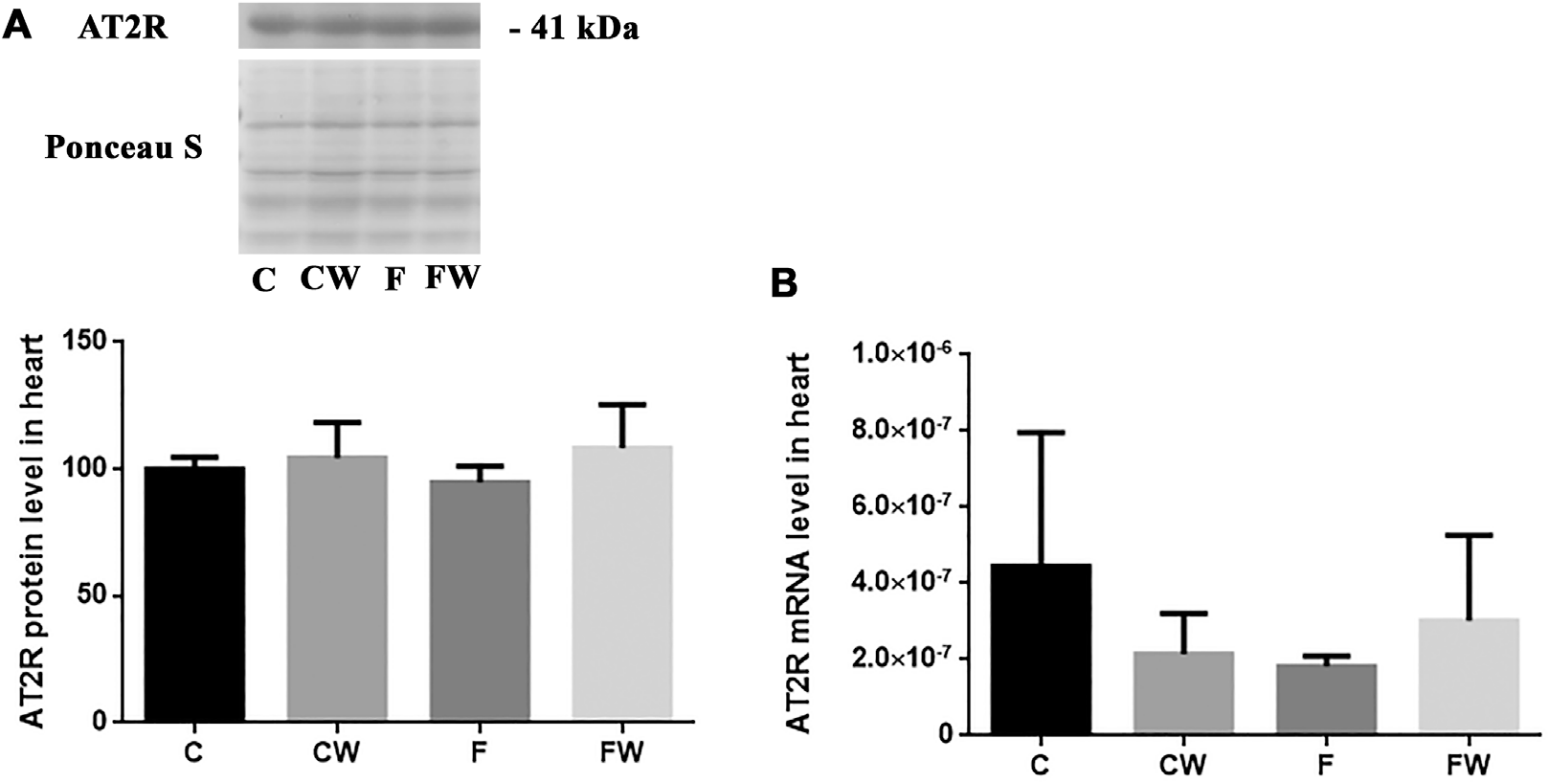
Effects of fructose-rich diet and walnut supplementation on **(A)** protein and **(B)** mRNA level of AT2R in heart of experimental rats. Values are means with standard deviations represented by vertical bars for 3 samples, each consisting of 3 pooled hearts of the same group. C—animals on standard laboratory chow, F—animals fed a fructose-rich diet, CW—animals on standard laboratory chow and walnut supplementation, FW—fructose-fed animals on walnut supplementation.

The level (protein or mRNA) of two main RAS receptors, AT1R (Figures 3A,B) and AT2R (Figures 4A,B), which mediate Ang II actions, remained unchanged after both treatments, alone or in combination.

### Effects of the fructose-rich diet and walnut supplementation on the expression of NF-κB in the heart of male rats

**FIGURE 5:**
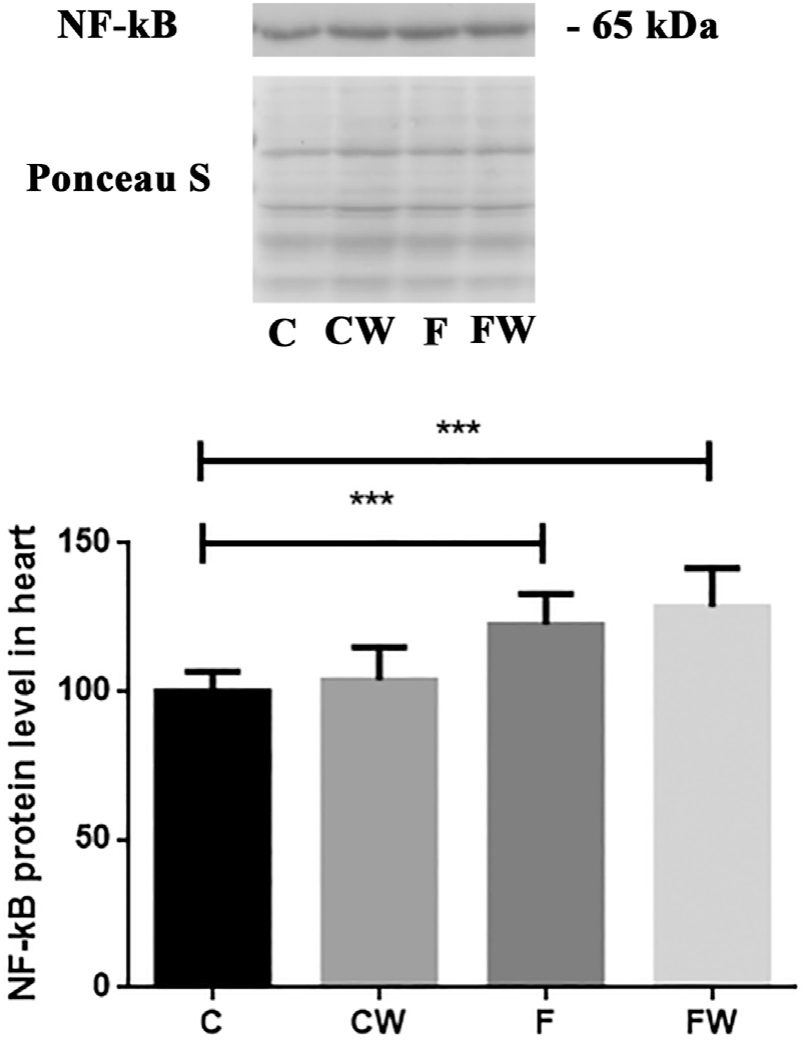
Effects of fructose-rich diet and walnut supplementation on NF-κB protein level in heart of experimental rats. Values are means with standard deviations represented by vertical bars for 3 samples, each consisting of 3 pooled hearts of the same group. C—animals on standard laboratory chow, F—animals fed a fructose-rich diet, CW—animals on standard laboratory chow and walnut supplementation, FW—fructose-fed animals on walnut supplementation, ***p < 0.001.

The results demonstrated a significant effect of fructose (*p* < 0.001) on the NF-κB protein level in the heart (Figure 5). FRD significantly increased the NF-κB protein level in rat hearts compared to control animals (C vs. F, *p* < 0.001). Walnut treatment combined with FRD did not revert elevated NF-κB protein level thus, it remained significantly higher compared to control rats (C vs. FW, *p* < 0.001).

## Discussion

Metabolic disturbances manifested in fructose feeding as an elevated level of serum insulin and dyslipidemia, as well as increased body weight and adiposity, may be modified at the molecular level by walnut supplementation (Zec et al., 2020; Bošković et al., 2021; Stanišić et al., 2021).

The main novelty in the current study is a significant main effect of walnut supplementation on the ACE2 protein level in the heart, which is in line with previously published significant reduction of systolic blood pressure in the current animal model of MetS (Bošković et al., 2021). ACE2 deficiency was associated with modest systolic hypertension (Gurley et al., 2006) and ACE2 activators lowered blood pressure (Hernandez Prada et al., 2008) and improved cardiac function (Dong et al., 2012; Murca et al., 2012). Not only that ACE2 is an important regulator of heart function (Crackower et al., 2002) but also exerts anti-inflammatory properties (Kassiri et al., 2009). It was shown that systemic delivery of ACE2 decreased Ang II-induced hypertrophy and fibrosis (Huentelman et al., 2005) even those that are not present in ACE2 knockout mice (Crackower et al., 2002). In addition, along with anti-fibrotic effects of ACE2 therapy using an adeno-associated viral vector it was shown that it was associated with downregulation of proinflammatory cytokines, monocyte chemoattractant protein-1 and interleukin (IL)-6, and decreased of lipid peroxidation products in the liver (Mak et al., 2015).

Walnut, the ingredient widely used in human diets, has the highest amount of polyunsaturated omega-3 fatty acids (n-3 PUFAs) among nuts (Leung et al., 2021), which have antioxidant and anti-inflammatory/immunomodulatory effects on the heart (Gutiérrez et al., 2019; Oppedisano et al., 2020). Our findings about walnut consumption effects on ACE2 also coincide with the results of previous studies that identified omega-3 PUFA as ACE2 modulators. The study of Ulu and co-workers has shown that a diet rich in omega-3 PUFA attenuated inflammation in Ang II-dependent hypertension by upregulating ACE2 mRNA levels in the kidney (Ulu et al., 2013).

In the current study, we demonstrated a decrease in the AA/EPA ratio in the heart after walnut consumption. Recently, we also reported a walnut-associated decrease of plasma AA/EPA ratio in the same animal model of MetS (Bošković et al., 2021). The impact of PUFA supplementation on ACE2 levels in the MetS has not been investigated, yet. The results of previous studies that related PUFA or polyphenol compounds with ACE2 are scarce and not consistent. In murine adipocytes, DHA increased ACE2 mRNA expression (Gupte 2011), while incubation of porcine adipocytes with DHA reduced ACE2 mRNA expression (Tseng et al., 2010). In contrast to adipose tissue, administration of DHA did not show an influence on ACE2 expression in the heart and kidney (Gupte 2011). Discrepancies in previous results may be due to differences in animal models, cell species and doses, duration, and type of treatments. ACE2 entered the scientific spotlight during the COVID-19 pandemic, and the majority of studies performed during the previous few years targeted compounds that could inhibit recombinant human ACE2 (rhACE2) activity. Several polyphenol-containing plant extracts (Takahashi et al., 2015; Takahashi S et al., 2017; Liu et al., 2020) inhibited rhACE2 activity, *in vitro* yet, some of them inhibited ACE, as well (Takahashi et al., 2015). Recent results revealed that the beneficial effects of PUFAs are attributed to their inhibitory binding effects on viral RBD of spike protein to the hACE2 receptor (Goc et al., 2021). However, supplementation by walnuts, the n3-PUFA-rich foodstuff, in MetS have a protective effect on the heart through beneficial change in cardiac n6/n3 fatty acid ratio and associated increased ACE2 expression in the heart. Furthermore, consumption of walnuts resulted in a decrease of the relative weight of the heart (Boškovic et al., 2021). This suggests that a diet rich in walnuts has some antihypertrophic capacity. This effect can be also attributed to the omega-3 fatty acids that walnut contains. This assumption is in line with the findings of Toko et al. (2020) showing that omega-3 fatty acids ameliorate cardiac dysfunction induced by pressure overload. In addition, increased whole nut (peanuts, pine nuts, and almonds) consumption was associated with a decline in the probability of left ventricular hypertrophy (Park et al., 2021). The stimulation of the ACE2/Ang 1–7 axis of the RAS may represent a powerful therapeutic approach in the treatment of CVD. ACE2 overexpression by gene transfer prevented cardiac fibrosis (Díez-Freire et al., 2006). Moreover, oral administration of ACE2 activators improves the function and remodeling of the heart in rat diabetes models (Dong et al., 2012; Murca et al., 2012). Having in mind that a recent meta-analysis of randomized controlled trials confirmed that supplementation of n-3 PUFA reduces the risk of cardiac mortality, major adverse cardiovascular events, and myocardial infarction (Casula et al., 2020) it is clear that changes in habitual diet might have long term beneficial health effects. Previous studies have implied that either a prolonged fructose diet regime or the increased percentage of consumed fructose finally does result in cardiac hypertrophy (Patel et al., 2009; Zhang et al., 2016). Walnut supplementation after FRD in our model showed upregulation of beneficial ACE2 protein and retained the relative heart weight (heart weight relative to body weight), indicating an absence of hypertrophy and protection of the heart.

However, the mechanisms that more precisely explain the regulation of ACE2 are still mostly undefined. It was revealed that the expression of ACE2 could be controlled by the activity of SIRT1 (Clarke et al., 2014). As we showed that walnut supplementation upregulates both, SIRT1 protein level (Boškovic et al., 2021) and ACE2 protein level in the FRD rat heart, it supports the hypothesis of enhanced expression of ACE2 by SIRT1, in MetS.

Furthermore, the anti-inflammatory and cardioprotective effects of walnut supplementation in MetS are supported by a decrease in the AA/EPA ratio in the heart as a result of walnut consumption. Recently we reported a walnut-dependent decrease of plasma AA/EPA ratio (Boškovic et al., 2021), and herein we confirmed it in the heart tissue. The study of Leung et al. (2021) revealed that the walnut diet significantly upregulated ALA and EPA levels, while it downregulated AA in the heart tissue compared to the control. Although we saw a decrease of AA/EPA ratio in the walnut diet in control animals it was not significant. Similarly, in control animals, we only detected a trend toward a decrease of ACE after walnut consumption. The study of Gencoglu et al. also indicated that there was no difference between the control and the walnut oil (WO) groups fed with a standard diet on body and liver weight changes, metabolic health risk factors, oxidative stress markers (eNOS), and transcription factors (NF-κB and Nrf2) as well as NADPH oxidase (p22phox) and SIRT1. On the other hand, they demonstrated that WO supplementation could alleviate the adverse impacts of both high carbohydrate (HCD) and high-fat diet (HFD) in rats (Gencoglu et al., 2020). As recently shown walnut diet did not affect inflammation-related gene expressions IL-6, IL-1β, and tumor necrosis factor *α* (TNF-α) compared to the control (Leung et al., 2021). Even though effects in controls still show some discrepancies, many studies underscore the promising potential of walnuts in both prevention and treatment of the MetS.

In addition, the mechanism of molecular actions should be further investigated. As expected, FRD elevated the protein level of NF-κB but 6-week long walnut consumption did not attenuate NF-κB mediated metabolic syndrome-related inflammation. Zhang et al. demonstrated that 12-week long oral administration of edible oils with a low n-6/n-3 PUFA ratio attenuated osteoarthritis-induced inflammation and progression via inhibiting the NF-κB pathway (Zhang et al., 2020). Prolonged consumption of walnuts might reduce the expression of NF-κB in the heart after FRD, as it has been shown that treatment with EPA or DHA reduced LPS-stimulated expression of proinflammatory cytokines in human macrophages, as well as NF-κB activity (Weldon et al., 2007). In our model of FDR walnut supplementation didn’t revert NADPH oxidase 4 (Nox4) change, also (Boškovic et al., 2021). Upregulated ACE in FRD could lead to higher levels of Ang II, which could increase NADPH oxidase 4 (Nox4) production (Harrison et al., 2003). We don’t propose that walnut-associated upregulation of ACE2 could completely attenuate effects of ACE or related Ang II effects but, these mechanisms are dependent on AT1R, which expression was not affected in the current study. This also may explain the absence of changes in the heart mass, i.e. cardiac hypertrophy.

One of this study’s limitations is the use of commercial antibodies, as their specificity was questioned and discussed previously (Gautron, 2019), particular for angiotensin receptors (Hafko et al., 2013). AT1R antibody that we used has been used in many studies, among which one confirmed its specificity in HEK293 cells (Czikora et al., 2015). In addition, a recent study developed a monoclonal anti-ATR001 antibody which they confirmed in comparison to the anti-AT1R antibody used in our study (Zhang et al., 2019). To partially overcome this limitation, we confirmed the same band for AT2R in the aorta (data not shown) and we have also provided mRNA data for AT1R and AT2R, which consistent with protein quantification shows no effects of walnut supplementation on receptor expression. We might also consider a limitation of our study to be the lack of measurement of cardiac levels of other RAS components including Ang II and Ang (1-7). The comparable activity levels of ACE and ACE2 within the heart should also be determined in further studies given the strong expression of ACE after FRD in our study. It is known that ACE could cleave the Ang (1-7) thus, reducing its beneficial actions. However, in a comprehensive review of the RAS was noted that it is more common in circulation and kidneys (Chappell, 2016). Envisioning of the processes within RAS becomes even more complex upon introducing Ang-(1-9), another ACE2 metabolite with a role in the cardiovascular system, which could also be cleaved by ACE (McKinney et al., 2014). It is clear that RAS is a rather complex system with expressed tissue specificity and an array of enzymes with different affinities for both effector proteins and receptors.

In conclusion, the 6-week walnut supplementation had a beneficial effect on the ACE2 protein level and AA/EPA ratio, the cardioprotective components, thus contributing to the protection of the heart in MetS. The stimulation of the ACE2/Ang 1–7 axis of the RAS may represent a powerful therapeutic approach in the treatment of cardiometabolic diseases, thus habitual walnut consumption emerges as a powerful lifestyle strategy. Moreover, as walnuts are a good source of proteins, arginine, polyphenols, and cations with antihypertensive properties, further investigation of nutritional ACE2 activators might have additional benefits to cardioprotective effects in combined approach with conventional RAS blockers (such as ACE inhibitors and AT1 receptor antagonist) in cardiovascular diseases. In the presence of AT1R blockade, the activation of the ACE2/Ang (1–7)/MasR axis contributes to the improvement of the myocardium (Sukumaran et al., 2012) and vascular remodeling (Savoia et al., 2020). Further work should additionally focus on the specifics of omega-3-rich walnut supplementation, in terms of heart tissue damage/reparation in relationship with dosing and duration of dietary walnut treatment.

## Data Availability

The original contributions presented in the study are included in the article/Supplementary Materials, further inquiries can be directed to the corresponding author.
