# Supplementary figures and images for: Walnut supplementation after fructose-rich diet is associated with a beneficial fatty acid ratio and increased ACE2 expression in the rat heart
Source: Front Physiol. 2022 Sep 21;13:942459. doi: 10.3389/fphys.2022.942459 (PMC9533082; doi:10.3389/fphys.2022.942459)

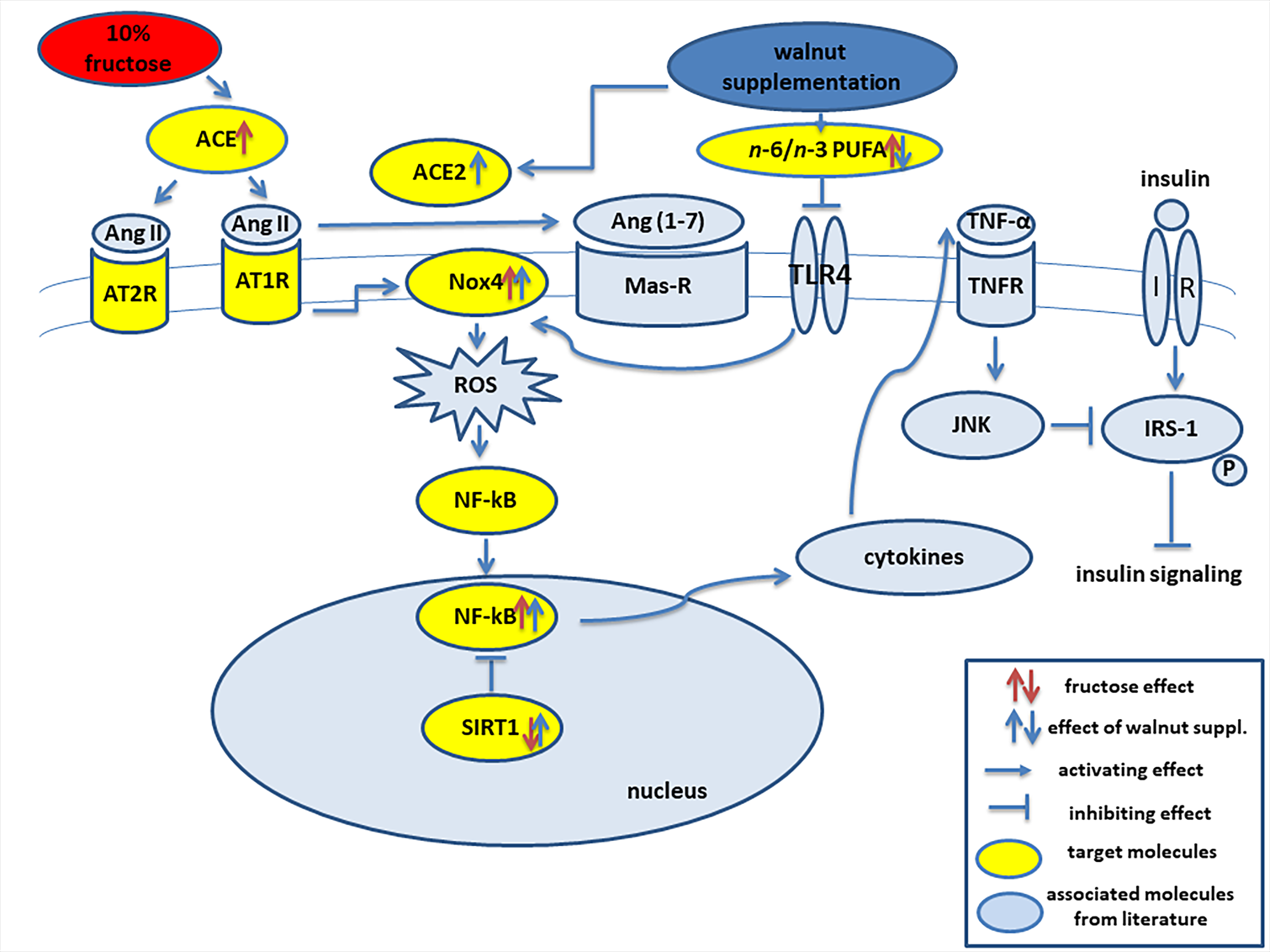

Supplement: Supplementary file 1 [file Image1.TIF]
